# Supplementary material for: Integrative Metabolomic and Transcriptomic Analysis Reveals the Mechanism of Specific Color Formation in Phoebe zhennan Heartwood
Source: Int J Mol Sci. 2022 Nov 5;23(21):13569. doi: 10.3390/ijms232113569 (PMC9658324; doi:10.3390/ijms232113569)
Supplement: Supplementary file 1 [file ijms-23-13569-s001.zip › Supplementary Figures.pdf]

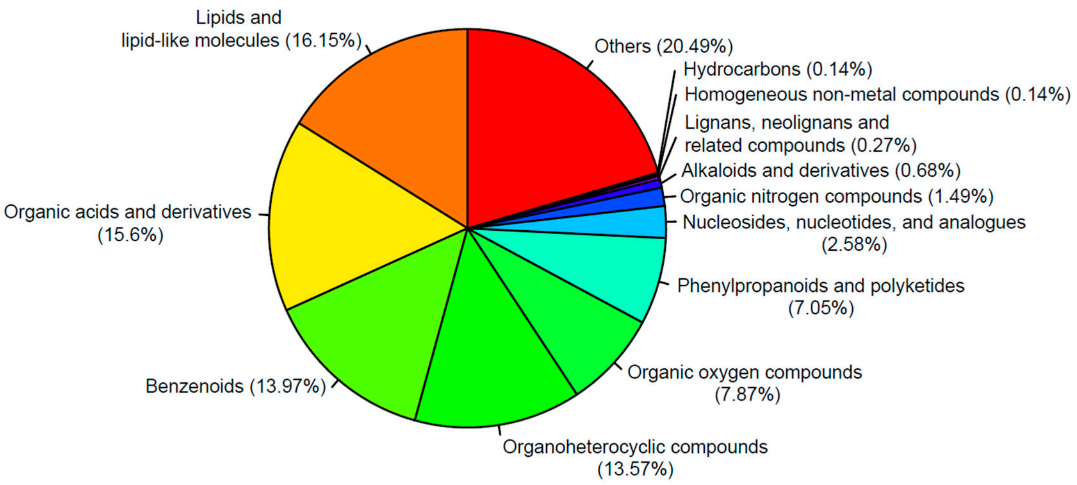

Figure S1. Classification of the identified metabolites annotated using the HMDB database

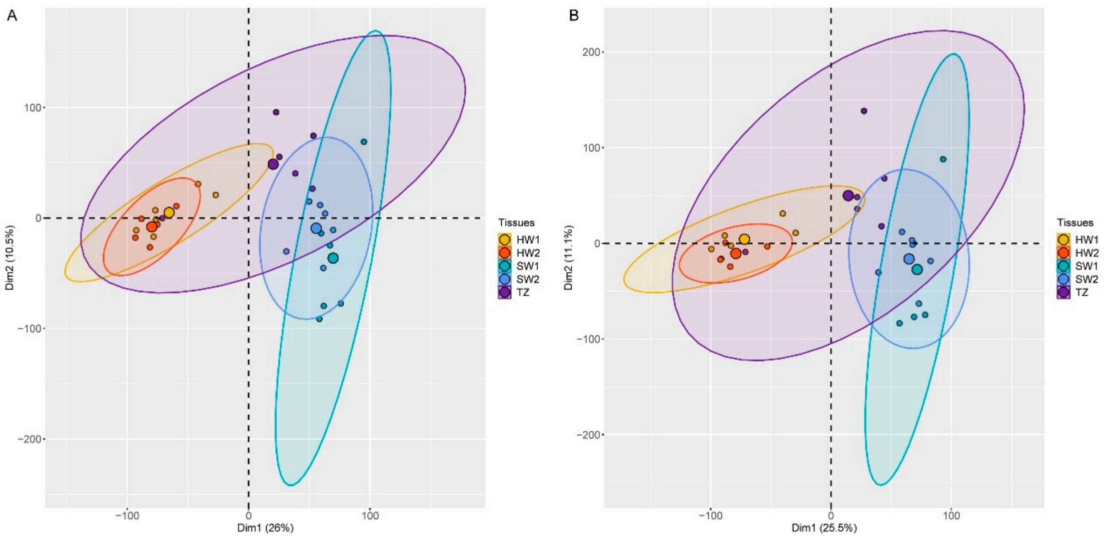

Figure S2. Principal component analysis (PCA) of metabolites in positive (A) and negative (B) models

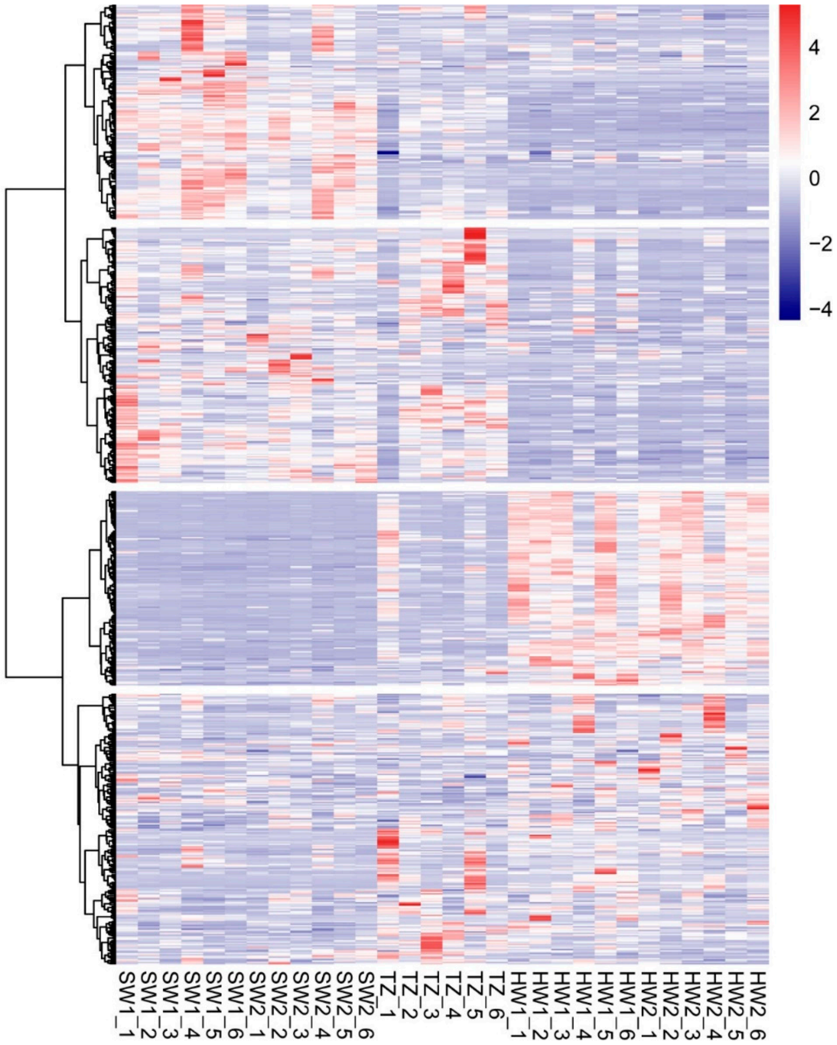

Figure S3. Hierarchical clustering of all identified metabolites among five tissues.

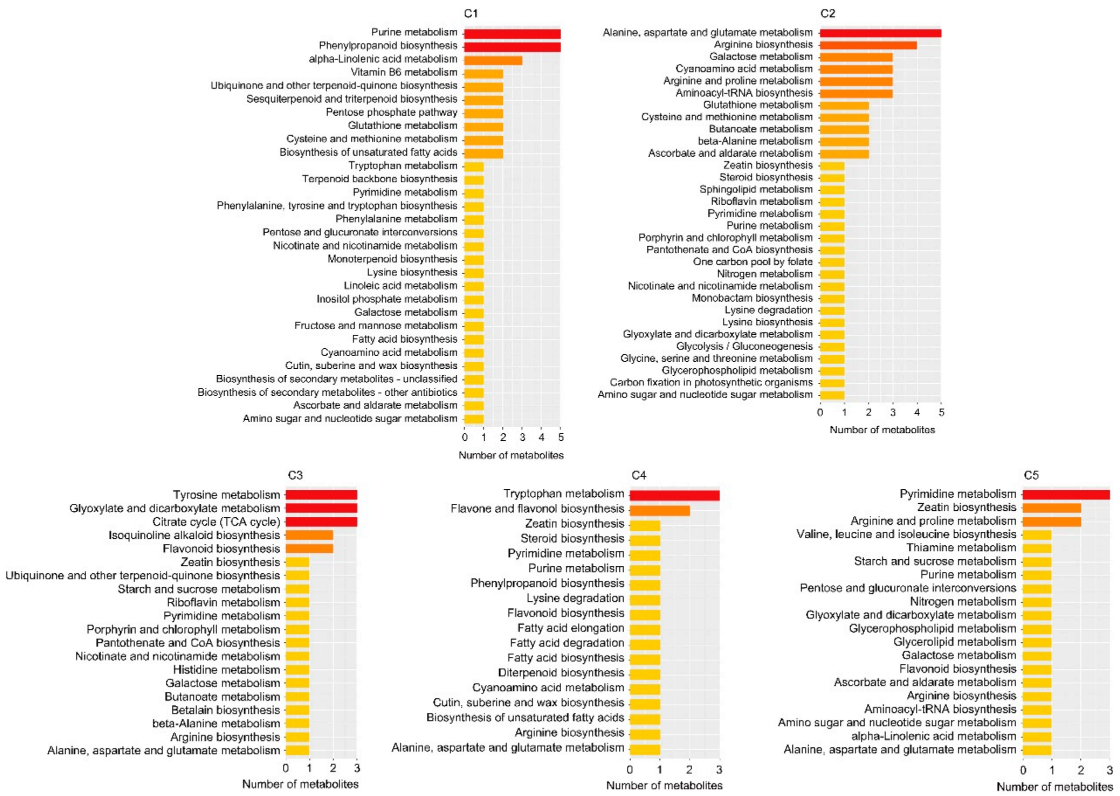

Figure S4. The classification of five clusters in the KEGG pathway

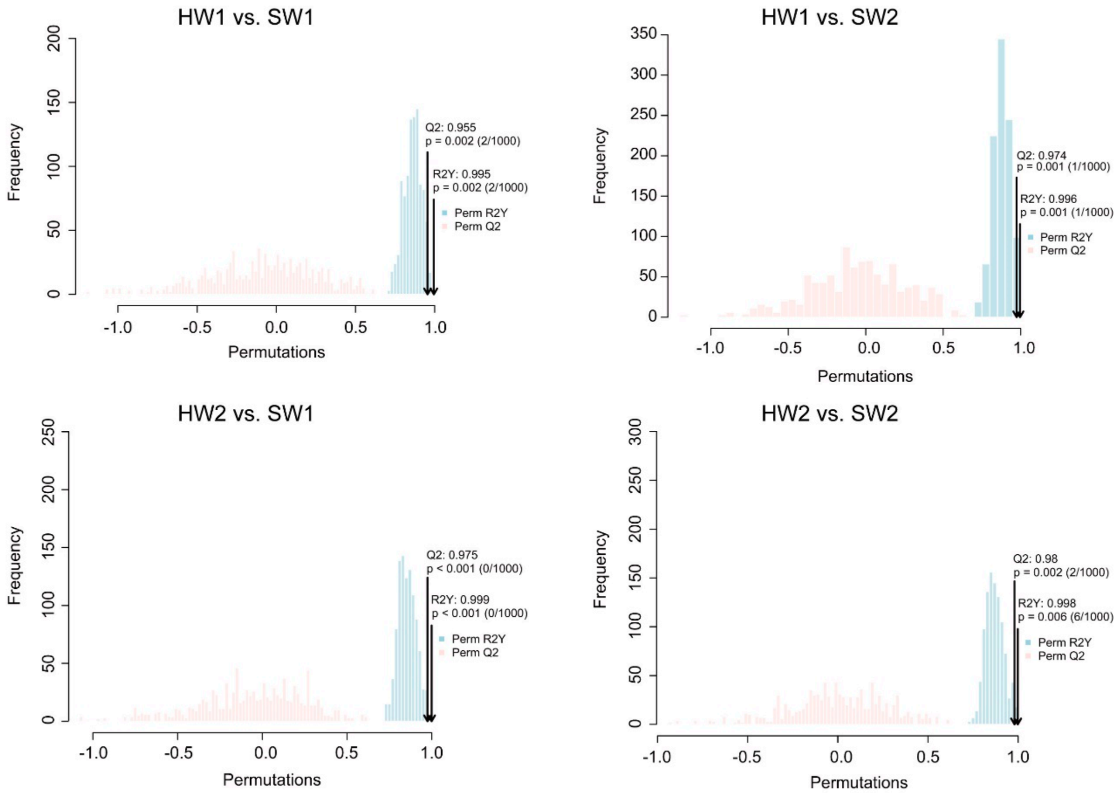

Figure S5. Permutation test plot with 1000 iterations. The abscissa represents the accuracy of the model, and the ordinate is the frequency of the model classification effect.

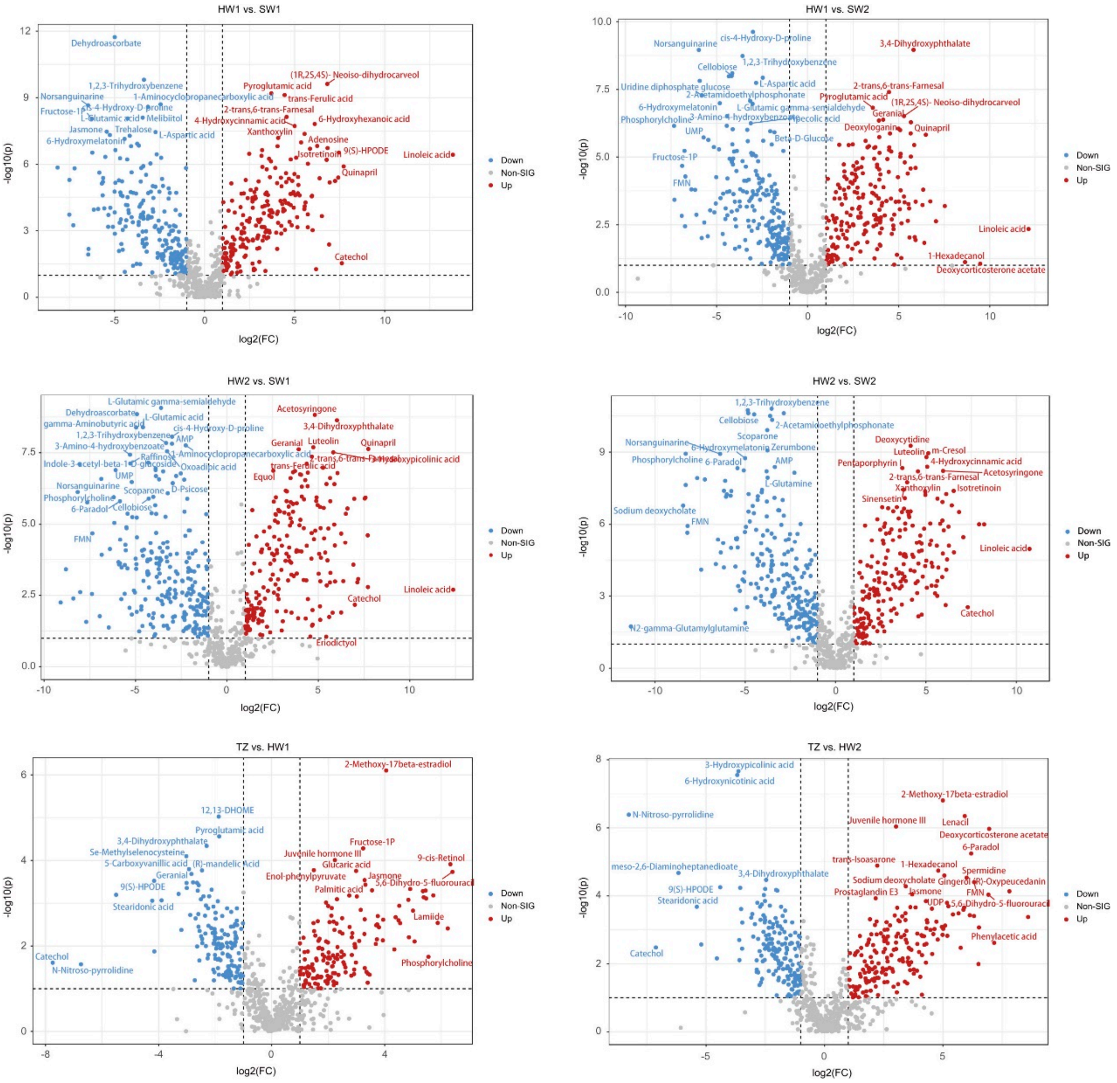

Figure S6. Volcano plot of differentially metabolites among sapwood, transition zone and heartwood.

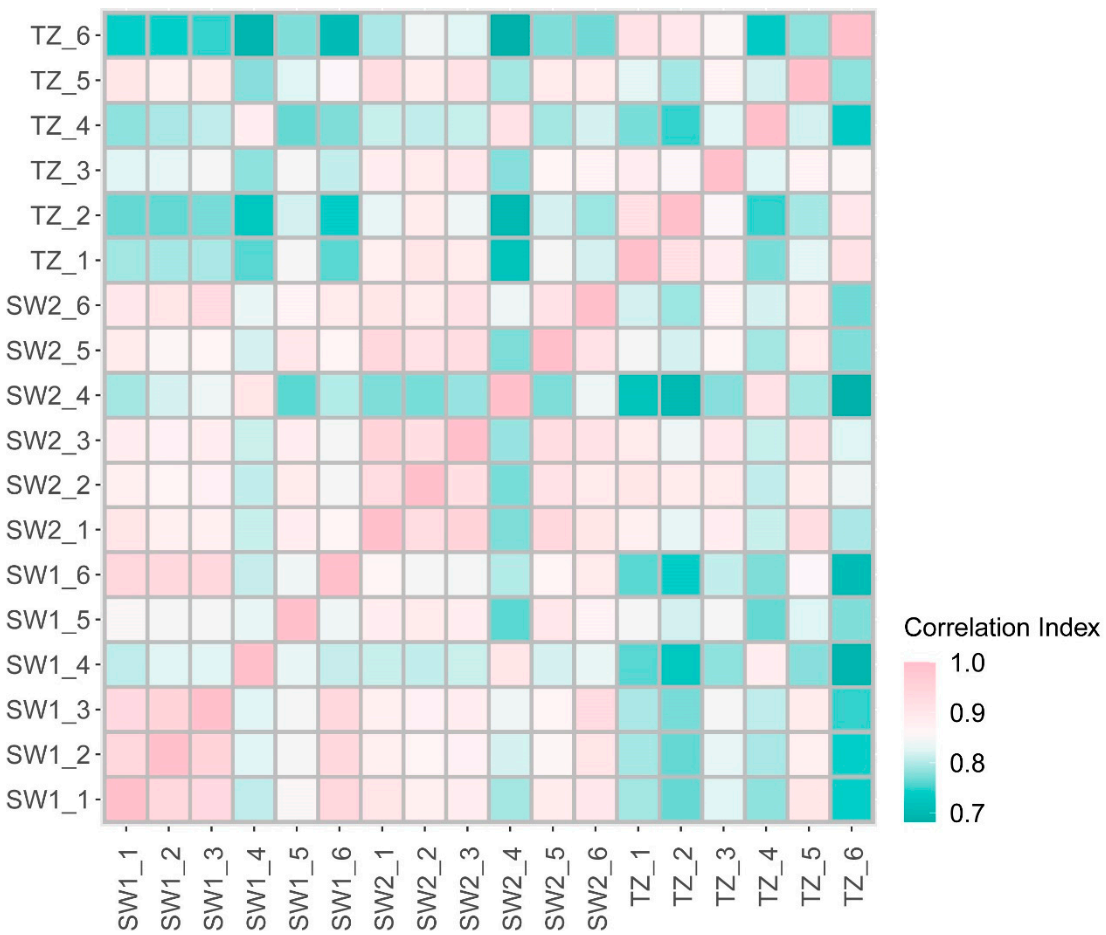

Figure S7. Pearson correlation analysis for all transcriptome samples.

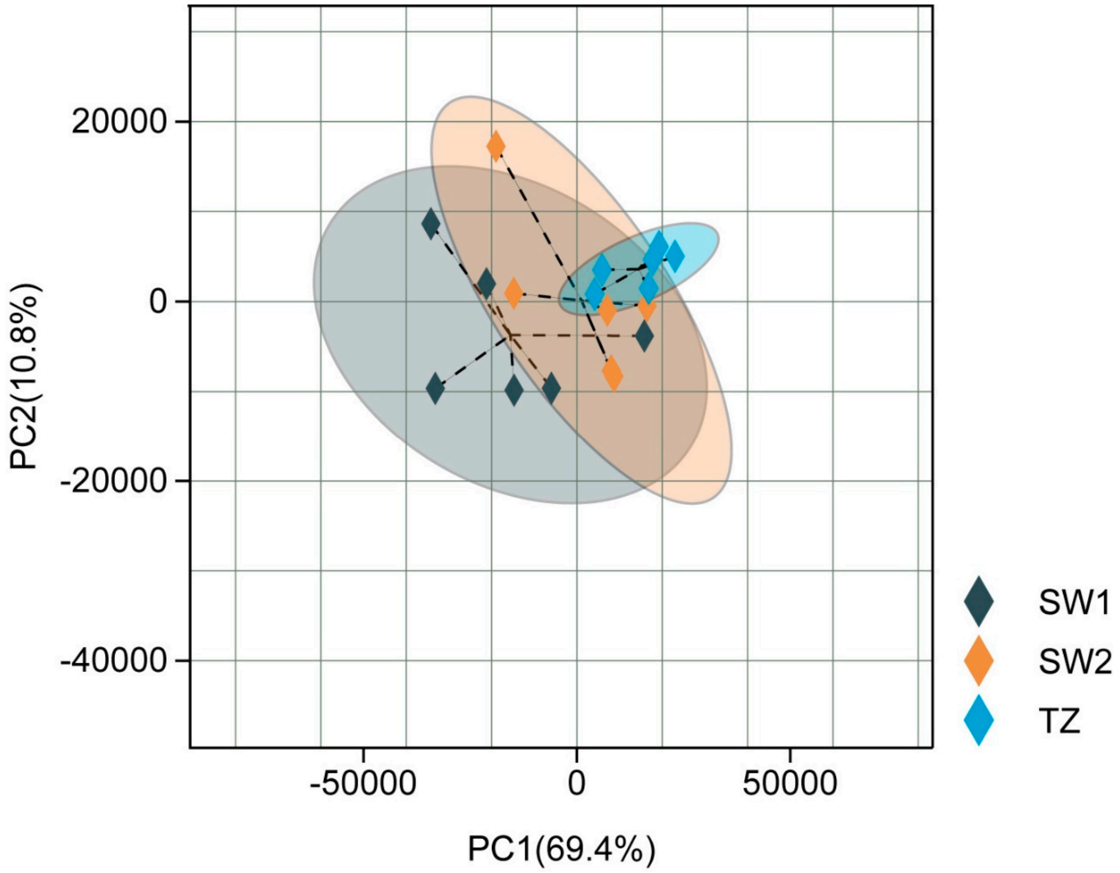

Figure S8. Principal component analysis (PCA) score plots for all transcriptome samples

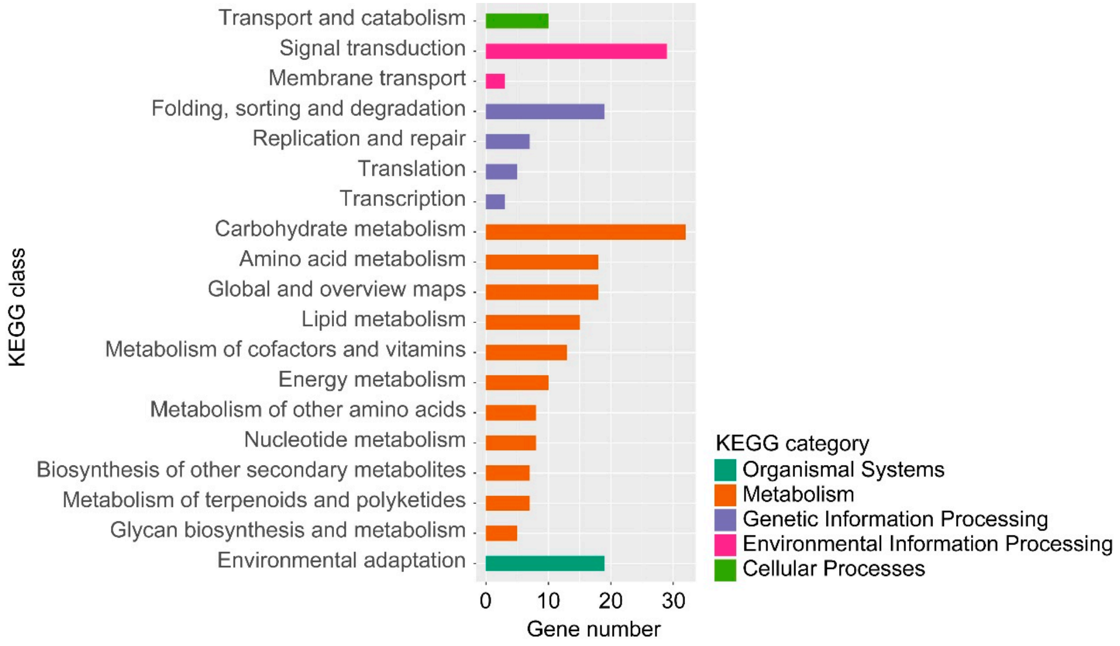

Figure S9. The KEGG category of 528 DEGs between sapwood and transition zone.

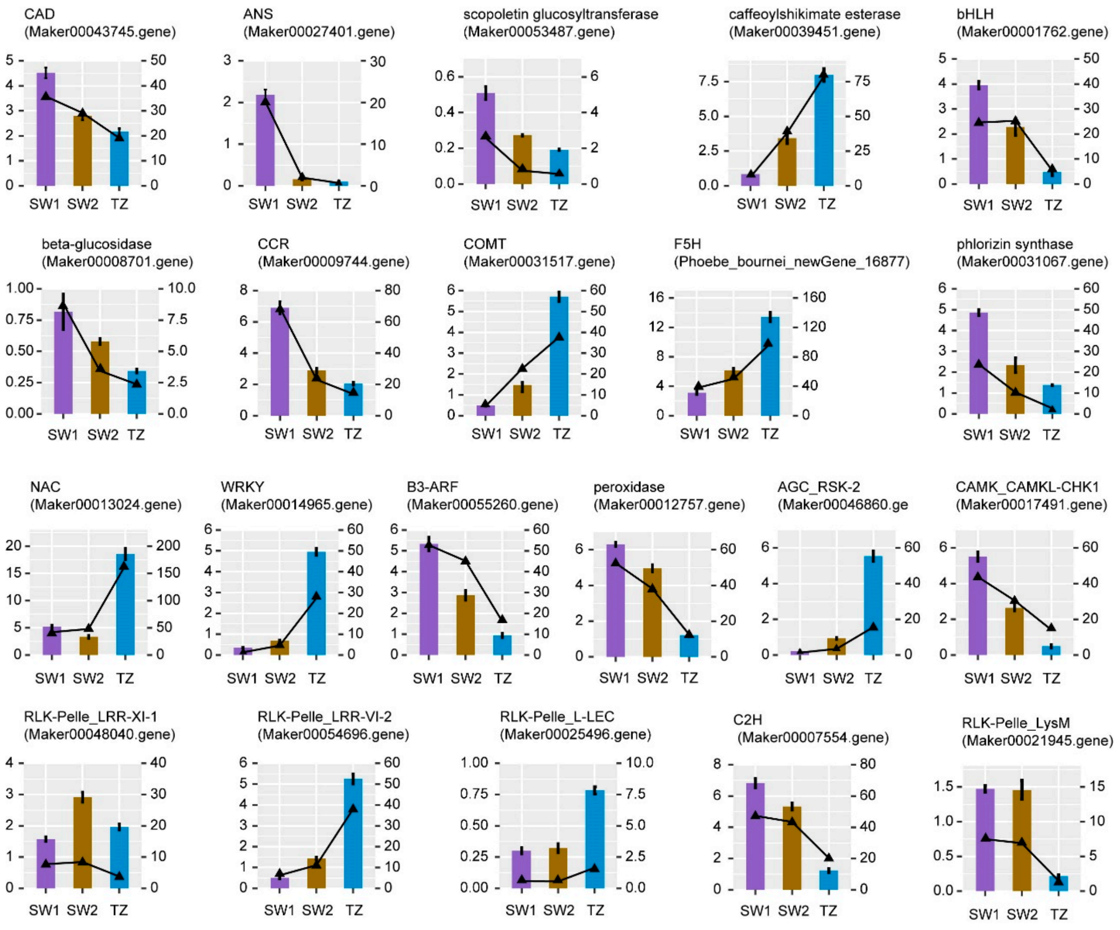

Figure S10. The RT-qPCR analysis of the relative expression level (left) and fragment per kilobase per million reads (FPKM) (right) of 21 enzyme genes and transcription factors in SW1, SW2 and TZ. The column showed the relative expression level, the line showed the FPKM value.

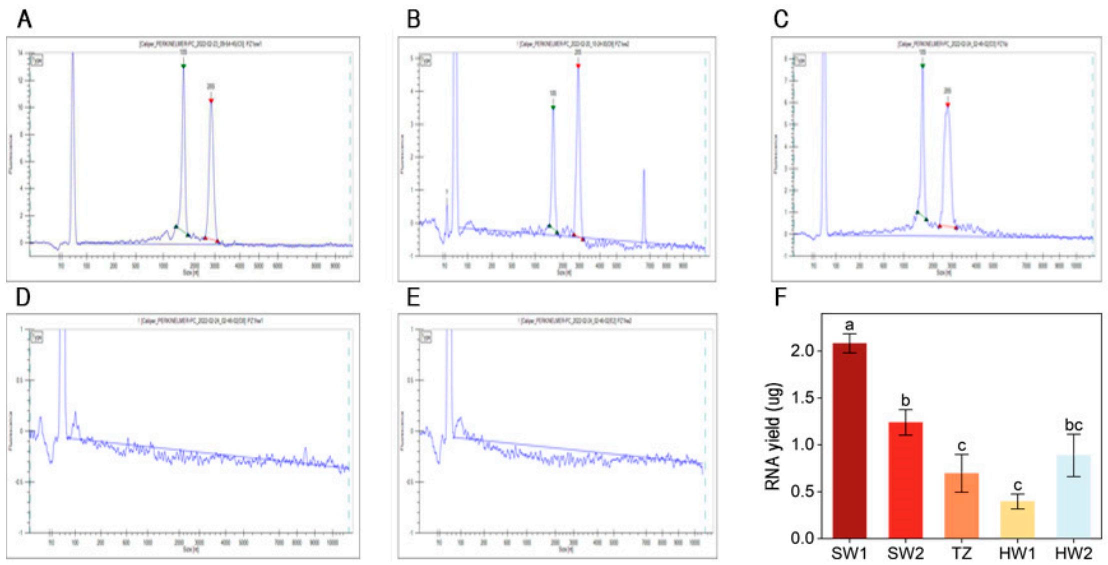

Figure S11. The production and quality of the total RNA from sapwood, transition zone and heartwood. (A-E) The results of integrity test of total RNA from outer sapwood (A, SW1), inner sapwood (B, SW2), transition zone (C, TZ), outer heartwood (D, HW1) and inner heartwood (E, HW2). (F) The production of total RNA in five tissues. The same letters indicated no significant difference between tissues at the level of 0.05, the different letters indicated significant difference between tissues at the level of 0.05.
